# Preoperative differentiation of retroperitoneal ganglioneuroma and schwannoma using an ultrasonography-based multivariable model and simplified score: development and single-center internal validation

**DOI:** 10.3389/fsurg.2025.1685442

**Published:** 2025-11-20

**Authors:** Haining Zheng, Meiying Gao, Jin Cui, Xiaoying Zhang, Wenjie Li, Xuemei Ma, Chaoyang Wen

**Affiliations:** 1 Department of Ultrasound, Peking University International Hospital, Beijing, China; 2 Department of Pathology, Peking University International Hospital, Beijing, China; 3 Department of Retroperitoneal Tumor Surgery, Peking University International Hospital, Beijing, China

**Keywords:** retroperitoneal tumor, ganglioneuroma, schwannoma, ultrasonographic features, predictive model, simplified scoring system, decision curve analysis

## Abstract

**Objective:**

This study aims to develop and internally validate a multivariable logistic regression model and a simplified scoring system, based on standardized ultrasonographic features, for the preoperative differentiation of retroperitoneal ganglioneuroma (GN) from schwannoma (SW), and to evaluate their discrimination, calibration, and clinical utility.

**Methods:**

We retrospectively included patients with retroperitoneal GN or SW confirmed by surgical pathology. Standardized ultrasonographic features were extracted, and candidate predictors were selected using least absolute shrinkage and selection operator (LASSO) regression, while retaining potential confounders (age, sex, lesion long diameter). A multivariable model was constructed, and a six-variable simplified score was derived. Discrimination [area under the curve (AUC)], calibration (intercept, slope, Brier score), and decision curve analysis (DCA) were evaluated using stratified fivefold cross-validation and bootstrap resampling (*B* = 2,000). Two task-oriented thresholds were predefined: R1 [rule-out, sensitivity (Se) ≥ 0.95] and S1 [standard diagnosis, specificity (Sp) ≥ 0.50].

**Results:**

A total of 74 patients were included (GN, 25, 33.8%; SW, 49, 66.2%). After optimism correction, the multivariable model achieved an AUC of 0.930, and the simplified score achieved an AUC of 0.917. Independent predictors included pelvic extraperitoneal location (*loc_pelvic* = 1), absence of cystic/necrotic change, and lower SD/LD ratio. For R1, the model threshold of 0.149 yielded Se = 0.960, Sp = 0.837, and negative predictive value (NPV) = 0.976; the score threshold of 0.206 yielded Se = 1.000, Sp = 0.592, and NPV = 1.000. For S1, the model threshold of 0.426 yielded Se = 0.920 and Sp = 0.939, and the score threshold of 0.594 yielded Se = 0.760 and Sp = 0.918.

**Conclusion:**

Both the multivariable model and the simplified score demonstrated excellent performance in differentiating GN from SW, suggesting potential value as rapid, interpretable tools for bedside use and in resource-limited settings. Their clinical utility should be confirmed through external validation and recalibration in multicenter, prospective cohorts and further enhanced through integration with multimodal imaging such as CT, MRI, and contrast-enhanced ultrasound (CEUS).

## Introduction

Ganglioneuroma (GN) and schwannoma (SW) are relatively common benign neurogenic tumors of the retroperitoneum. Although both entities are histologically benign, they differ markedly in biological behavior, preferred surgical approaches, and postoperative surveillance strategies. Consequently, accurate preoperative discrimination between GN and SW is essential for individualized surgical planning and optimizing long-term patient outcomes (1–3). Evidence from both multicenter and single-center cohort studies has described the imaging appearances, clinical management, and prognoses of GN (1, 4), emphasizing the real-world value of reliable preoperative identification. In contrast to malignant retroperitoneal sarcomas, these two benign tumors warrant distinctly different management pathways and are associated with substantially different prognostic profiles (5, 6). Although both are benign, GN typically follows an indolent clinical course after complete excision with lower surveillance intensity, whereas SW may exhibit higher local recurrence risk requiring closer postoperative surveillance and, in selected cases, wider *en bloc* resection margins. These distinctions motivate an accurate preoperative differentiation to individualize surgical planning and follow-up.

In contemporary clinical practice, contrast-enhanced computed tomography (CT) and magnetic resonance imaging (MRI) remain the cornerstone modalities for evaluating the origin, anatomical extent, and relationships of retroperitoneal tumors to adjacent structures (7–9). Recent advances in radiomics—exemplified by the multicenter RADSARC-R study published in *Lancet Oncology* in 2023 (10)—have demonstrated that CT-derived radiomics signatures can classify histologic subtypes and grades of retroperitoneal sarcomas with high accuracy. Nonetheless, such approaches depend on high-quality cross-sectional imaging and advanced post-processing platforms, which limit their feasibility in emergency, bedside, or resource-limited settings.

Ultrasonography, in contrast, offers real-time imaging, wide availability, low cost, and freedom from ionizing radiation, making it an indispensable tool for the initial assessment and follow-up of retroperitoneal masses (11, 12). Prior studies have shown that ultrasonography can depict key morphologic and vascular characteristics of neurogenic tumors—including tumor shape, margin definition, internal echotexture, cystic or necrotic change, posterior acoustic enhancement, and vascularity—which can aid in differentiating between pathologic subtypes and distinguishing benign from malignant lesions (12–15). For palpable or superficial soft tissue masses, ultrasonography is consistently recommended as the first-line imaging modality for triage, while MRI is reserved for complex or suspicious lesions to enable detailed characterization and staging (11, 12). Notably, the 2023 European Society of Musculoskeletal Radiology (ESSR) consensus introduced standardized interpretive criteria for adult soft tissue tumors, and the 2022 Society of Radiologists in Ultrasound (SRU) consensus by Jacobson et al. established uniform terminology and key interpretive points for superficial soft tissue mass evaluation. In the present study, we adopted these terminology frameworks to standardize the ultrasonographic assessment of retroperitoneal neurogenic tumors, thereby improving the reproducibility and consistency of feature interpretation.

On this basis, we developed and internally validated a multivariable logistic regression model grounded in a unified dictionary of ultrasonographic features to distinguish GN from SW preoperatively. From this model, we derived a simplified scoring system designed to be both interpretable and readily applicable at the bedside. The potential clinical utility of this approach warrants further evaluation in larger, multicenter cohorts.

## Methods

### Study design and ethical approval

This was a single-center retrospective observational study conducted in compliance with the Transparent Reporting of a multivariable prediction model for Individual Prognosis Or Diagnosis (TRIPOD) principles. The protocol was approved by the institutional ethics committee, with informed consent waived due to the retrospective use of anonymized imaging and pathology data.

### Study population

The patients who underwent surgical resection of a retroperitoneal tumor between December 2016 and November 2024 were identified from the institutional database. The inclusion criteria were as follows:

1. Histopathological diagnosis of GN or SW
2. Preoperative ultrasonographic images of sufficient quality for feature assessment
3. Complete clinical and pathological data

The exclusion criteria were as follows:

1. Concomitant malignant tumors
2. Missing key imaging planes or poor image quality
3. Imaging-to-surgery interval >3 months

### Ultrasonographic examination

All examinations were performed by radiologists with at least 5 years of abdominal ultrasonography experience, using a Philips iU Elite color Doppler system (1–5 MHz transducer). Standard transverse, longitudinal, and any additional planes required for lesion characterization were obtained and stored in the picture archiving and communication system (PACS). B-mode and color Doppler modes were applied, with scanning parameters adjusted to the patient’s habitus. Only cases with diagnostic-quality images, including transverse and longitudinal planes, were included; however, as a retrospective PACS review, we acknowledge that certain features (e.g., calcification) might not be captured if not present on the stored planes.

### Features and data preprocessing

A standardized ultrasonographic feature dictionary was used to ensure consistency. The recorded variables included the following:

- Location: pelvic extraperitoneal (presacral/iliac fossa; yes/no). For clarity, the retroperitoneum inherently includes the pelvic portion; here, “pelvic extraperitoneal” was used as a shorthand for presacral/iliac fossa location.
- Shape: regular vs. irregular
- Margin: well-defined vs. ill-defined
- Internal echotexture: hypoechoic vs. non-hypoechoic
- Cystic/necrotic change: present vs. absent
- Calcification: present vs. absent
- Posterior acoustic enhancement: present vs. absent
- Tumor vessel encasement sign: present vs. absent
- Vascularity: present vs. absent on color Doppler
- Sex: male/female
- Age: years
- Tumor diameters: long diameter (LD), short diameter (SD), and SD/LD ratio

Two radiologists independently evaluated images, and disagreements were resolved by consensus. All features were coded as binary or continuous variables as appropriate. Data completeness was verified (no missing data). Both radiologists were blinded to histopathological results during feature assessment.

### Model development

Feature selection was performed using least absolute shrinkage and selection operator (LASSO) logistic regression, retaining potential confounders (age, sex, LD). The selected variables were entered into a multivariable logistic regression to obtain adjusted odds ratios.

A simplified scoring system was derived from the final binary predictors, assigning one point per predictor (total score 0–6). A sensitivity analysis compared equal-weight and coefficient-weighted scoring for diagnostic performance and calibration.

### Model evaluation and validation

Model performance was assessed in terms of the following:

- Discrimination: receiver operating characteristic (ROC) analysis with area under the curve (AUC) calculation
- Calibration: intercept, slope, and Brier score estimation; stratified calibration with 10 equal-frequency bins
- Uniform shrinkage: adjustment of coefficients using the optimism-corrected calibration slope (16)
- Clinical utility: decision curve analysis (DCA) to evaluate net benefit
- Internal validation: stratified fivefold out-of-fold (OOF) cross-validation for the simplified score
- Additional validation: nested cross-validation and bootstrap (.632+) resampling to assess model robustness

### Threshold strategies

Two task-oriented thresholds were predefined:

- R1 (rule-out): sensitivity ≥0.95, maximizing specificity under this constraint
- S1 (standard diagnosis): specificity ≥0.50, maximizing the Youden index under this constraint

### Statistical analysis

Unless otherwise specified, values were reported to three decimal places; *P* < 0.001 were reported as “<0.001.” Se, Sp, positive predictive value (PPV), and negative predictive value (NPV) were calculated with 95% confidence intervals using the Wilson method. Normality of continuous variables was tested; appropriate parametric or non-parametric tests were applied. Categorical variables were analyzed using the *χ*^2^ test or Fisher's exact test. All analyses were performed in Python (v3.9) with standard statistical and plotting libraries.

## Results

### Patient characteristics

A total of 74 patients were included: 25 (33.8%) with GN and 49 (66.2%) with SW. Baseline demographic and ultrasonographic characteristics are summarized in Table 1. A study flow description has been added, reporting numbers potentially eligible, excluded with reasons, and included (see Supplementary eFigure S2).

**Table 1:** Baseline characteristics of patients with GN vs. SW.

Variable	SW	GN	*P*-value
Sex	33 (67.3%) 16 (32.7%)	15 (60.0%) 10 (40.0%)	0.610
Age (years)	46.00 ± 15.56 [50.00 (33.00, 59.00)]	32.92 ± 15.01 [27.00 (23.00, 43.00)]	<0.001
Symptoms	5 (10.2%) 5 (10.2%) 21 (42.9%) 6 (12.2%) 12 (24.5%)	2 (8.0%) 0 (0.0%) 20 (80.0%) 1 (4.0%) 2 (8.0%)	0.035
Number of lesions	43 (87.8%) 6 (12.2%)	24 (96.0%) 1 (4.0%)	0.411
Location	25 (51.0%) 5 (10.2%) 19 (38.8%)	1 (4.0%) 1 (4.0%) 23 (92.0%)	<0.001
LD (cm)	7.72 ± 3.66 [7.60 (5.30, 9.60)]	12.21 ± 5.41 [11.40 (7.70, 15.30)]	<0.001
SD (cm)	5.37 ± 2.58 [4.90 (3.90, 6.40)]	5.34 ± 2.64 [4.80 (3.50, 6.40)]	0.749
SD/LD ratio	0.72 ± 0.16 [0.73 (0.58, 0.85)]	0.46 ± 0.17 [0.42 (0.38, 0.54)]	<0.001
Shape	10 (20.4%) 39 (79.6%)	18 (72.0%) 7 (28.0%)	<0.001
Margin	3 (6.1%) 46 (93.9%)	13 (52.0%) 12 (48.0%)	<0.001
Internal echo	48 (98.0%) 0 (0.0%) 1 (2.0%)	19 (76.0%) 6 (24.0%) 0 (0.0%)	0.001
Cystic/necrosis	24 (49.0%) 25 (51.0%)	23 (92.0%) 2 (8.0%)	<0.001
Calcification	43 (87.8%) 6 (12.2%)	19 (76.0%) 6 (24.0%)	0.317
Posterior echo	21 (42.9%) 27 (55.1%) 1 (2.0%)	4 (16.0%) 21 (84.0%) 0 (0.0%)	0.046
Blood flow	16 (32.7%) 8 (16.3%) 1 (2.0%) 24 (49.0%)	5 (20.0%) 3 (12.0%) 2 (8.0%) 15 (60.0%)	0.396
Vessel encasement	46 (93.9%) 3 (6.1%)	12 (48.0%) 13 (52.0%)	<0.001

GN, ganglioneuroma; SW, schwannoma; LD, long diameter; SD, short diameter; SD/LD, short-to-long diameter ratio. *P*-values were calculated using *t*-test or Mann–Whitney *U*-test for continuous variables, and chi-square test or Fisher's exact test for categorical variables as appropriate. Location was dichotomized as pelvic extraperitoneal (presacral/iliac fossa) vs. non-pelvic for modeling. Symptoms were categorized as follows: (1) asymptomatic/incidental finding; (2) abdominal pain/discomfort; (3) low back pain/discomfort; (4) lower-limb pain/discomfort; (5) palpable mass without discomfort.

### Univariate analysis

All odds ratios (ORs) were calculated with GN as the outcome event. Binary features significantly associated with a higher likelihood of GN included ill-defined margin (OR = 16.6, *P* < 0.001), tumor vessel encasement sign (OR = 16.6, *P* < 0.001), and irregular shape (OR = 10.0, *P* < 0.001). Features favoring SW (OR < 1 for GN) included pelvic extraperitoneal location (OR = 0.04, *P* < 0.001), cystic/necrotic change (OR = 0.083, *P* < 0.001), and posterior acoustic enhancement (OR = 0.254, *P* = 0.006).

**Figure 1:**
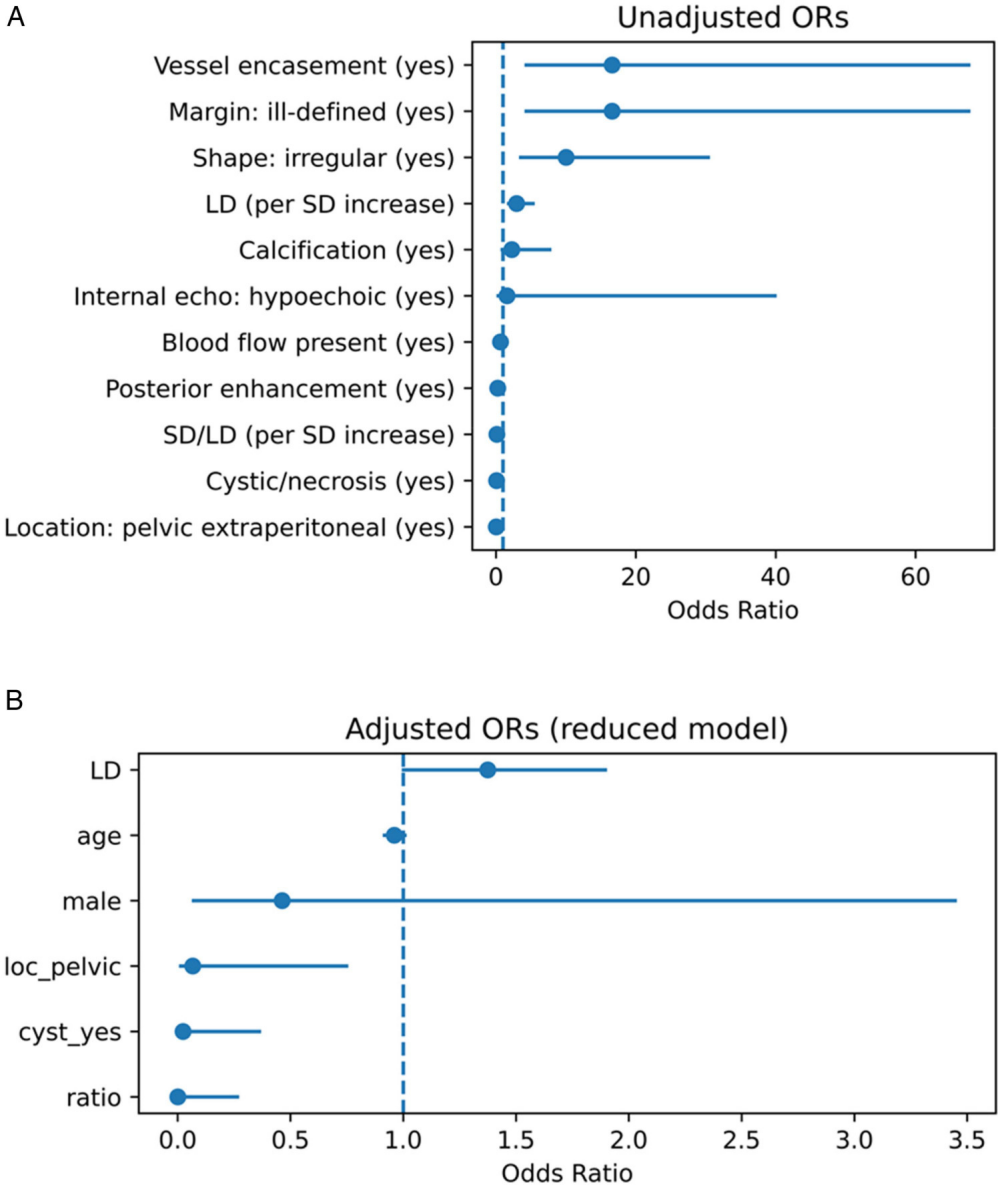
**(A)** Univariate associations between ultrasound features and the diagnosis of GN vs. SW. Forest plot showing unadjusted odds ratios (ORs) and 95% confidence intervals (CIs) from univariate analyses. Binary variables are shown as ORs; continuous variables are shown per unit increase or Hodges–Lehmann median difference. GN, ganglioneuroma; SW, schwannoma; LD, long diameter; SD, short diameter; SD/LD, short-to-long diameter ratio. **(B)** Adjusted multivariable associations between ultrasound features and the diagnosis of GN vs. SW. Forest plot showing adjusted odds ratios (ORs) and 95% confidence intervals (CIs) from the reduced multivariable logistic regression model. Variables included were selected by LASSO and adjusted for potential confounders (age, sex, long diameter). GN, ganglioneuroma; SW, schwannoma; LD, long diameter; SD, short diameter; SD/LD, short-to-long diameter ratio.

Among continuous variables, the median LD was significantly larger in GN [Hodges–Lehmann (HL) difference ≈ +3.8 cm, *P* ≈ 3.5 × 10^−4^], whereas the SD/LD ratio was significantly lower in GN (HL difference ≈ −0.269, *P* ≈ 1.9 × 10^−7^). Details are shown in Figure 1A (binary predictors) and Figure 1B (continuous predictors), as well as Supplementary eTables S1 and S2.

### Multivariable analysis

LASSO regression selected three major predictors (location, cystic/necrotic change, SD/LD), which were entered into multivariable logistic regression together with potential confounders (LD, age, sex).

The final model included six variables:

- Pelvic extraperitoneal location (OR = 0.067, *P* = 0.029; favoring SW)
- Absence of cystic/necrotic change (OR = 0.023, *P* = 0.008; favoring GN)
- SD/LD per +1 unit (OR = 0.00067, *P* = 0.017; lower ratio favoring GN)
- LD (borderline effect, OR = 1.375, *P* = 0.055)
- Age and sex (non-significant)

Details are provided in Supplementary eTable S3A; scaled and shrunken effects are summarized in Supplementary eTables S3B and C, respectively.

### Model performance

**Figure 2:**
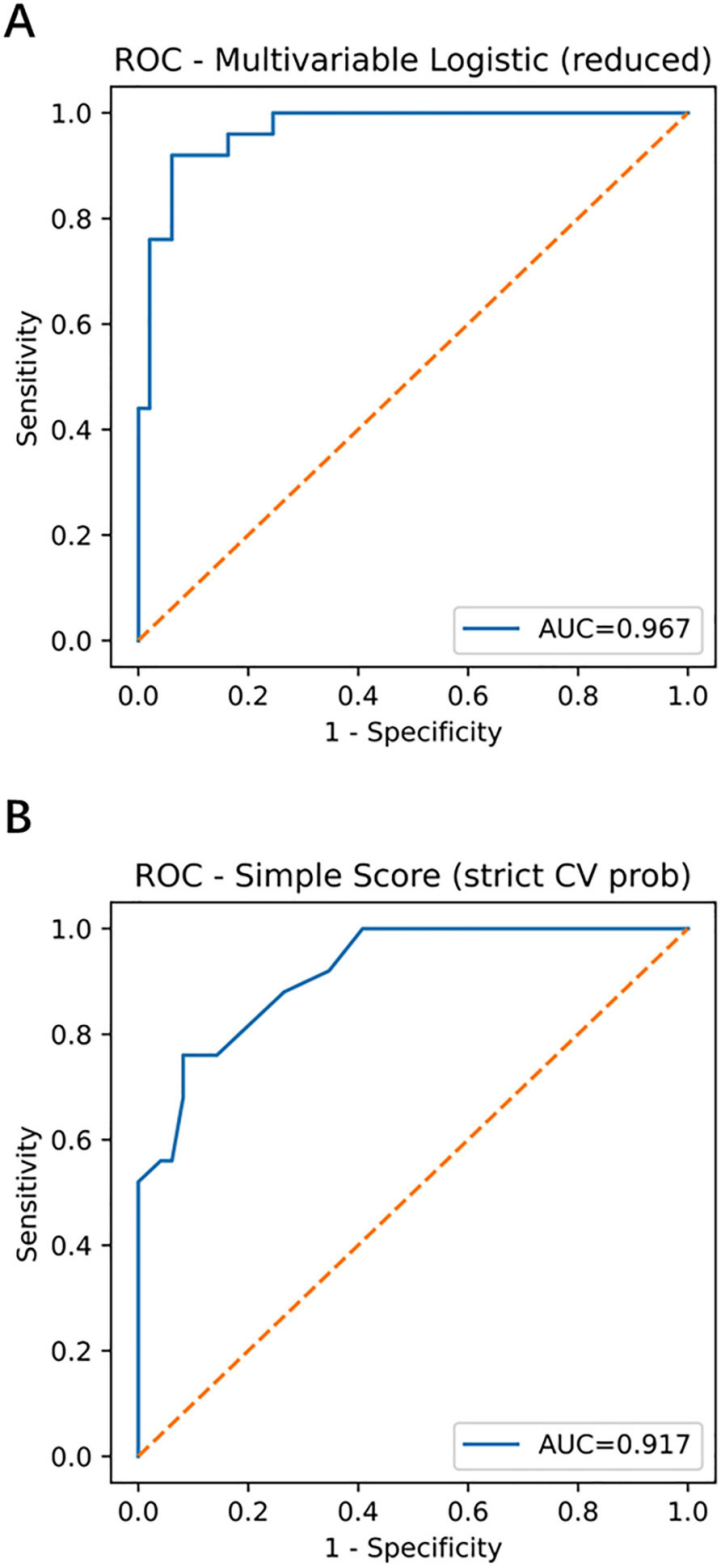
Receiver operating characteristic (ROC) curves of the multivariable model and simplified score. **(A)** Multivariable model with apparent AUC = 0.967 (95% CI, 0.926–0.995) and optimism-corrected AUC = 0.930. **(B)** Simplified score with stratified fivefold cross-validation AUC = 0.917 (95% CI, 0.846–0.968). ROC, receiver operating characteristic; AUC, area under the curve; CI, confidence interval; GN, ganglioneuroma; SW, schwannoma.

The multivariable model demonstrated strong discrimination: apparent AUC = 0.967 (95% CI, 0.926–0.995), optimism-corrected AUC = 0.930. The simplified score (fivefold cross-validation) yielded an AUC = 0.917 (95% CI, 0.846–0.968) (Figures 2A,B; Tables 2 and 3).

**Table 2:** Discrimination of the multivariable model (AUC, 95% CI).

Model	AUC	95% CI (lower)	95% CI (upper)
Multivariable model	0.967	0.926	0.995

AUC, area under the curve; CI, confidence interval.

**Table 3:** Performance of the simplified score (stratified fivefold CV AUC, 95% CI).

Model	AUC	95% CI (lower)	95% CI (upper)
Simple score (strict fivefold CV prob)	0.917	0.846	0.968

AUC, area under the curve; CI, confidence interval; CV, cross-validation.

#### Calibration

**Figure 3:**
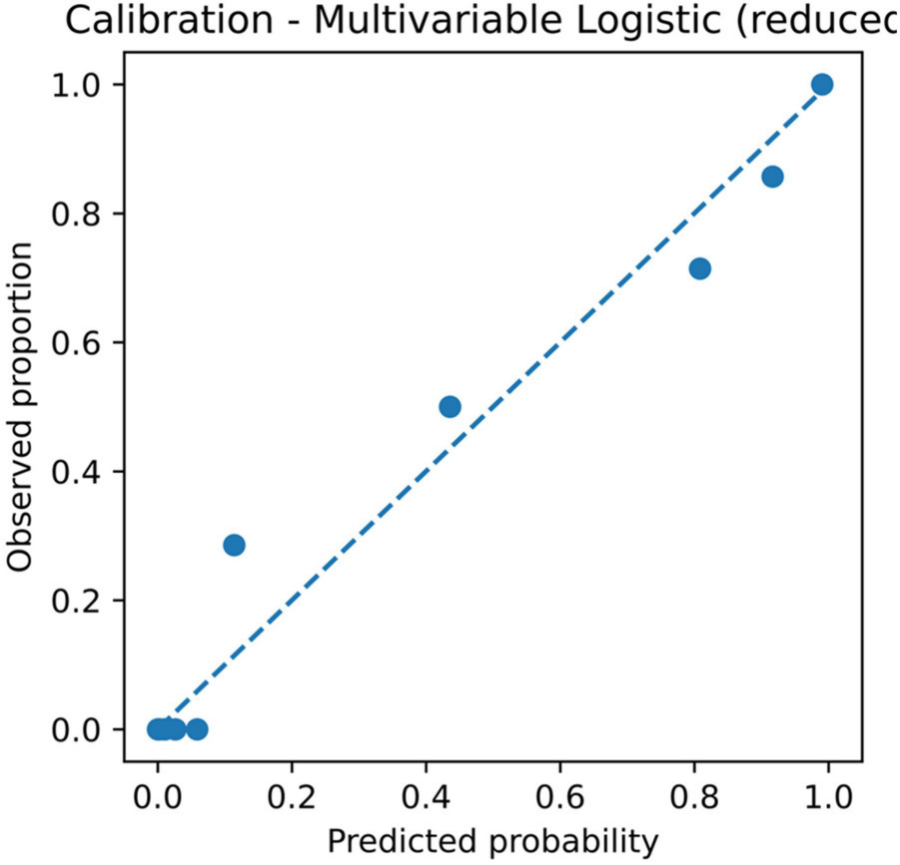
Calibration plot of the multivariable logistic regression model. The plot shows the relationship between predicted probabilities and observed proportions. Apparent slope = 1.000 and Brier score = 0.068; optimism-corrected slope = 0.442 and Brier score = 0.101, based on 2,000 bootstrap resamples. CI, confidence interval; GN, ganglioneuroma; SW, schwannoma.

For the multivariable model, the apparent slope was 1.000 with a Brier score of 0.068; the optimism-corrected slope was 0.442 with a Brier score of 0.101. The simplified score, using fivefold out-of-fold calibration, had an intercept of −0.003, a slope of 0.483, and a Brier score of 0.100 (Figure 3; Table 4). The optimism-corrected calibration slope of 0.442 indicates that the apparent predictions were over-extreme; substantial shrinkage and two-step recalibration (adjusting the intercept and then the slope) are recommended prior to external application.

**Table 4:** Calibration metrics: intercept, slope, and Brier score.

Phase	Intercept	Slope	Brier score
Apparent	0.000	1.000	0.068
Optimism-corrected	0.040	0.442	0.101

CI, confidence interval. Calibration metrics were estimated using 2,000 bootstrap resamples.

Additional performance metrics:

- Fivefold out-of-fold calibration (reduced six-variable model): intercept = −0.003, slope = 0.483, Brier score = 0.100, AUC = 0.904.
- L1-regularized logistic regression with nested cross-validation: outer AUC = 0.956 ± 0.030, Brier score = 0.088 ± 0.050.
- Bootstrap (.632+): AUC = 0.948, Brier score = 0.113.

#### Precision–recall analysis

Average precision (AP) was 0.937 for the multivariable model and 0.915 for the simplified score (Supplementary eFigure S1 and eTable S4). Notably, Supplementary eFigure S1 illustrates the PR curve for the multivariable model and shows the simplified score, highlighting both tools' robustness in imbalanced data contexts. Stratified calibration and extended calibration metrics are presented in Supplementary eTables S5A and B.

### Decision curve analysis

**Figure 4:**
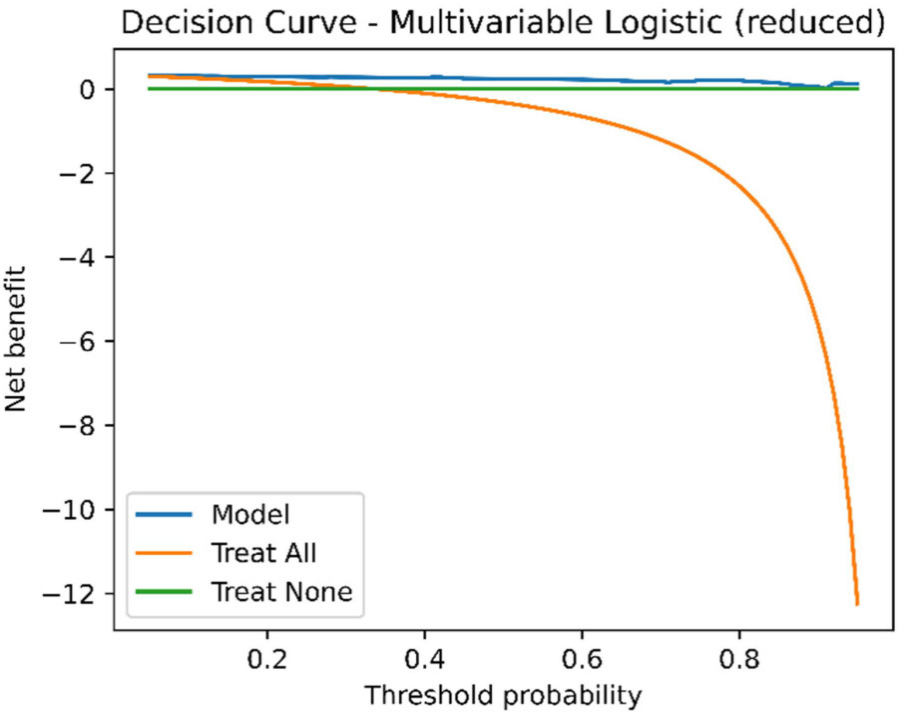
Decision curve analysis (DCA) of the multivariable model. Net benefit is plotted against threshold probability. The multivariable model shows a higher net benefit than both treat-all and treat-none strategies across threshold probabilities from 0.2 to 0.8. DCA, decision curve analysis; GN, ganglioneuroma; SW, schwannoma.

Across the threshold probability range of 0.20–0.80, the multivariable model consistently provided higher net benefit than the treat-all and treat-none strategies (Figure 4), supporting its potential clinical utility in preoperative decision-making.

### Threshold-based diagnostic performance

For clinical application, two predefined thresholds were evaluated:

- R1 (rule-out): high sensitivity (≥0.95) for excluding GN
- S1 (standard diagnosis): specificity ≥ 0.50 for confirming GN

Model R1/S1 thresholds: 0.149/0.426

Score R1/S1 thresholds: 0.206/0.594

Model performance (Table 5):

- R1: Se = 0.960, Sp = 0.837, PPV = 0.750, NPV = 0.976
- S1: Se = 0.920, Sp = 0.939, PPV = 0.885, NPV = 0.958

Score performance (Table 6):

**Table 5:** Task-oriented thresholds (model): R1 (Se ≥ 0.95) and S1 (Sp ≥ 0.50) (for preoperative assessment, exploratory).

Strategy	Threshold	Sensitivity	Specificity	PPV	NPV
S1: Sp ≥ 0.50	0.426	0.920	0.939	0.885	0.958
R1: rule-out (Se ≥ 0.95)	0.149	0.960	0.837	0.750	0.976

PPV, positive predictive value; NPV, negative predictive value; Se, sensitivity; Sp, specificity.

**Table 6:** Task-oriented thresholds (simplified score): R1 (Se ≥ 0.95) and S1 (Sp ≥ 0.50) (for preoperative assessment, exploratory).

Strategy	Threshold	Sensitivity	Specificity	PPV	NPV
S1: Sp ≥ 0.50	0.594	0.760	0.918	0.826	0.882
R1: rule-out (Se ≥ 0.95)	0.206	1.000	0.592	0.556	1.000

PPV, positive predictive value; NPV, negative predictive value; Se, sensitivity; Sp, specificity.

- R1: Se = 1.000, Sp = 0.592, PPV = 0.556, NPV = 1.000
- S1: Se = 0.760, Sp = 0.918, PPV = 0.826, NPV = 0.882

Confusion matrices are provided in Supplementary eTable S6, illustrating the trade-off between sensitivity and specificity for clinical decision-making. These results align with the model's intended clinical roles described in the Discussion, where R1 supports high-sensitivity exclusion and S1 enables more confident confirmation. Calculator parameters and an example are summarized in Supplementary eTables S11 and S12.

### Extended score analysis

- Equal-weight vs. weighted scoring: AUC = 0.931 vs. 0.940; minimal difference, equal-weight retained for bedside use (Supplementary eTable S7).
- Score-to-probability mapping: For example, a score of 3 corresponds to an estimated probability of 0.206, 4 points ≈ 0.708, and 5 points ≈ 0.957. This mapping enables direct translation of a patient's score into an interpretable probability of GN, facilitating risk communication and clinical decision-making, and allowing individualized probability estimates (see Supplementary eTable S8), as further discussed in the section on clinical applicability.

## Discussion

### Key findings and underlying biological mechanisms

In this study, we developed and internally validated a multivariable logistic regression model and a simplified scoring system based on standardized ultrasonographic features to differentiate retroperitoneal GN from SW. Both tools demonstrated excellent discrimination, calibration, and clinical net benefit (apparent AUC = 0.967; optimism-corrected AUC = 0.930; simplified score cross-validated AUC = 0.917; Figure 2 and Supplementary eTable S9), with calibration slopes and Brier scores indicating robust model performance. Independent predictors in the final model included pelvic extraperitoneal location (*loc_pelvic* = 1; OR = 0.067, *P* = 0.029; favoring SW), absence of cystic/necrotic change (OR = 0.023, *P* = 0.008; favoring GN), and lower SD/LD ratio (OR = 0.00067 per +1 unit, *P* = 0.017; lower ratios favoring GN). LD showed a borderline effect (*P* = 0.055), whereas age and sex were not significant (Supplementary eTables S3A,C).

These findings have clear imaging–pathologic correlations:

1. Histologic/degenerative mechanisms

SW often contains Antoni B regions with marked degenerative changes (cystic degeneration, hemorrhage, myxoid change), producing hypoechoic cystic areas with possible posterior enhancement on B-mode ultrasonography; GN has a denser, more mature stroma, with a lower incidence of cystic change (8, 9, 17).

1. Geometric ratio characteristics

GN typically extends longitudinally along the sympathetic chain or nerve axis, producing a longer LD and narrower SD (lower SD/LD ratio); SW grows in an expansile manner, resulting in a more rounded or ovoid transverse section (2, 3, 18, 19). Moreover, pelvic retroperitoneal SWs likely originate from pelvic plexus nerves and tend to expand centrifugally within confined spaces, which is consistent with their more rounded morphology and with our finding that pelvic retroperitoneal location independently predicts SW.

Compared with most prior studies that relied on descriptive features or MRI-based models (AUC typically 0.85–0.90), our approach—integrating a unified ultrasonographic feature dictionary and the SD/LD ratio—achieved higher discrimination while using an accessible, non-ionizing modality. These structure–function correspondences likely underpin the stability of our model and strengthen its interpretability. In contrast to CT/MRI radiomics pipelines that require high-end imaging quality and dedicated post-processing, ultrasonography offers portability, timeliness, and bedside availability; our standardized ultrasonographic feature dictionary leverages these strengths while maintaining interpretability.

### Clinical translation of threshold strategies

We defined two task-oriented thresholds (Tables 5 and 6, Supplementary eTable S10):

- R1 (rule-out, Se ≥ 0.95): model threshold = 0.149 (Se = 0.960, NPV = 0.976); score threshold = 0.206 (Se = 1.000, NPV = 1.000), enabling zero false negatives in-sample.
- S1 (standard diagnosis, Sp ≥ 0.50): model threshold = 0.426 (Se = 0.920, Sp = 0.939, PPV = 0.885, NPV = 0.958); score threshold = 0.594 (Se = 0.760, Sp = 0.918).

In rapid triage within ultrasound departments, R1 could identify low-probability cases suitable for follow-up or de-escalation, reducing unnecessary advanced imaging, whereas S1 could flag moderate-to-high-probability cases for further MRI characterization or preoperative planning. Decision curve analysis confirmed higher net benefit than treat-all or treat-none strategies across clinically relevant ranges (20, 21). These roles align directly with our results, where R1 achieved maximal sensitivity and S1 balanced specificity with predictive value. However, these findings remain exploratory and require prospective multicenter validation. These thresholds require prospective multicenter validation before widespread adoption.

### Complementarity of B-mode and contrast-enhanced ultrasound

B-mode ultrasonography and contrast-enhanced ultrasound (CEUS) often yield concordant macroscopic findings (e.g., SW more frequently shows cystic change and heterogeneous enhancement), but CEUS uniquely assesses microvascular perfusion. CEUS–pathology correlation studies in retroperitoneal SW have demonstrated early-phase moderate-to-high enhancement patterns consistent with Antoni A/B vascular characteristics (15).

Following EFSUMB non-hepatic CEUS guidelines (22), integrating CEUS into our R1/S1 framework could refine risk stratification: CEUS might enhance specificity in equivocal or atypical cases, particularly in hypervascular SW subtypes, while R1/S1 thresholds provide structured decision-making for broader application. Future work should explore multimodal integration of CEUS parameters with standardized ultrasonographic features.

### Molecular and embryologic perspectives

From a molecular and developmental standpoint:

- **SOX10**: A neural crest differentiation marker, diffusely positive in SW, reflecting stable Schwannian differentiation and correlating with its frequent degenerative changes (23).
- **GD2**: Highly expressed in immature neuroblastic tumors but low or absent in mature GN, suggesting potential roles in molecular imaging or targeted contrast agents for morphologically ambiguous retroperitoneal neurogenic tumors (24).
- **Embryologic pathway**: GN distribution follows neural crest migration patterns, often extending “*en bloc*” along the sympathetic chain, which explains its lower SD/LD ratio and tendency to abut but not invade major vessels (25).

Linking these molecular profiles to ultrasonographic features may pave the way for multi-omic predictive models that combine imaging phenotypes with tissue biomarkers. Integration of these molecular profiles with ultrasonographic phenotypes could further improve model interpretability and precision imaging.

## 5 Limitations and future directions

This single-center retrospective study had a limited sample size [*N* = 74; GN = 25; events per variable (EPV) ≈ 4], which may introduce selection and spectrum bias. Some predictors, such as LD and sex, had wide confidence intervals, highlighting the need for larger datasets to confirm effect stability. Despite LASSO selection, bootstrap optimism correction, and uniform shrinkage, the limited sample size (*N* = 74; GN = 25; EPV ≈ 4) implies residual risk of overfitting and statistical imprecision, warranting cautious interpretation and external validation. Generalisability may be limited by single-center case-mix, operator experience, and device/vendor differences; multicenter prospective validation across diverse settings is warranted.

To mitigate overfitting, we applied bootstrap optimism correction (AUC reduced from 0.967 to 0.930; Brier score increased from 0.068 to 0.101; calibration slope = 0.442) and uniform shrinkage (Figure 3, Supplementary eTables S9 and S3C). Before external application, we recommend two-step recalibration (adjust intercept *a* and then slope *b*) (16) and recalculation of PPV/NPV based on local prevalence. We provide executable Excel calculators (Supplementary Data Sheets S1 and S2) and comma-separated values (CSV) recalibration examples (Supplementary Dataset S1) to support reproducibility.

Future studies should recruit multicenter, prospective cohorts including non-surgical cases, leverage semi-automated or AI-assisted ultrasonographic feature extraction, and evaluate the integration of CEUS and molecular data. These strategies may enhance model generalizability and promote clinical adoption.

This study developed and internally validated a multivariable logistic regression model (AUC = 0.930) and a simplified scoring system (AUC = 0.917) based on standardized ultrasonographic features to differentiate retroperitoneal GN from SW. Both tools demonstrated excellent discrimination, calibration, and clinical net benefit. The three core predictors—absence of cystic/necrotic change, lower SD/LD ratio, and pelvic extraperitoneal location—have clear imaging–pathologic underpinnings. These findings suggest that the proposed model and score could serve as rapid, interpretable tools for bedside and resource-limited settings. External validation in multicenter, prospective cohorts, ideally incorporating multimodal imaging features, is warranted to enhance diagnostic performance in complex or morphologically overlapping cases.

## Data Availability

The original contributions presented in the study are included in the article/Supplementary Material; further inquiries can be directed to the corresponding authors.
