# Supplementary material for: Preoperative differentiation of retroperitoneal ganglioneuroma and schwannoma using an ultrasonography-based multivariable model and simplified score: development and single-center internal validation
Source: Front Surg. 2025 Nov 20;12:1685442. doi: 10.3389/fsurg.2025.1685442 (PMC12675405; doi:10.3389/fsurg.2025.1685442)
Supplement: Supplementary file 1 [file Supplementaryfile1.zip › eFigure 1 and eTables.docx]

**eFigure 1. Precision–Recall (PR) curves of the multivariable model and the simplified score.**

Panel A: multivariable model (shrinkage‑adjusted predicted probabilities). Panel B: simplified score (5‑fold out‑of‑fold predicted probabilities). Positive class = ganglioneuroma (GN=1). Average precision (AP) values are 0.937 and 0.915, respectively; 95% CIs for AP are reported in eTable 4. PR curves complement ROC analysis under class imbalance; curves closer to the top‑right indicate better performance. Abbreviations: PR, precision–recall; AP, average precision; GN, ganglioneuroma; SW, schwannoma; OOF, out‑of‑fold.


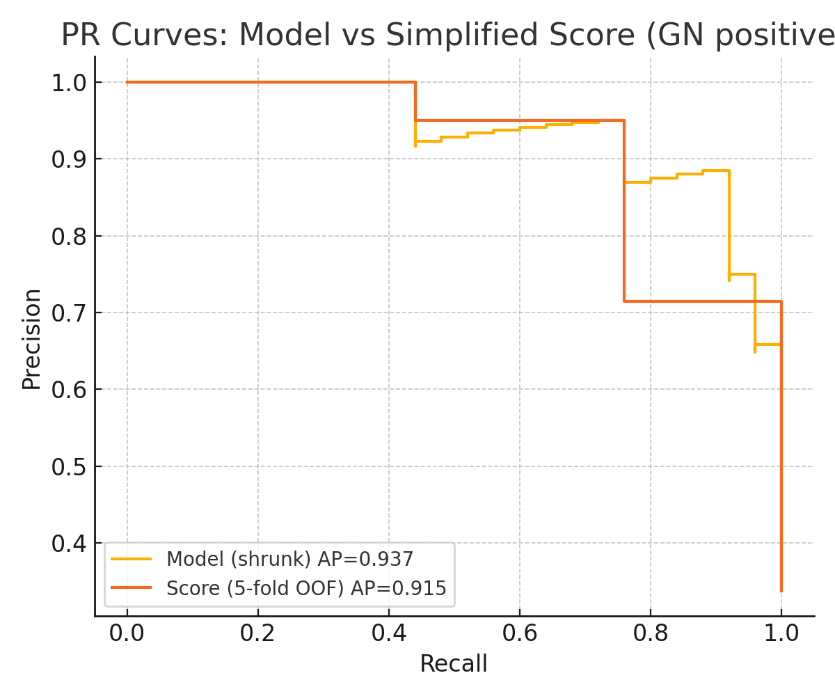


**eTable 1. Univariate Binary Associations of Ultrasound Features with GN vs SW**

Abbreviations: OR, odds ratio; CI, confidence interval; GN, ganglioneuroma; SW, schwannoma.

| Variable | OR (unadj) | 95%CI low | 95%CI high | P |
| --- | --- | --- | --- | --- |
| Location: pelvic extraperitoneal (yes) | 0.040 | 0.005 | 0.319 | <0.001 |
| Shape: irregular (yes) | 10.029 | 3.286 | 30.605 | <0.001 |
| Margin: ill-defined (yes) | 16.611 | 4.068 | 67.834 | <0.001 |
| Cystic/necrosis (yes) | 0.083 | 0.018 | 0.393 | <0.001 |
| Posterior enhancement (yes) | 0.254 | 0.076 | 0.851 | 0.021 |
| Vessel encasement (yes) | 16.611 | 4.068 | 67.834 | <0.001 |
| Blood flow present (yes) | 0.640 | 0.241 | 1.700 | 0.369 |
| Calcification (yes) | 2.263 | 0.646 | 7.929 | 0.317 |
| Internal echo: hypoechoic (yes) | 1.577 | 0.062 | 40.127 | 1.000 |

**eTable 2. Univariate Continuous Associations of Ultrasound Features with GN vs SW**

Abbreviations: HL diff, Hodges–Lehmann median difference; CI, confidence interval; GN, ganglioneuroma; SW, schwannoma.

| Variable | HL_diff (GN - SW) | 95%CI low | 95%CI high | P |
| --- | --- | --- | --- | --- |
| LD (cm) | 3.800 | 1.800 | 6.500 | <0.001 |
| SD/LD | -0.269 | -0.352 | -0.195 | <0.001 |

**eTable 3. Multivariable logistic regression: adjusted odds ratios (95% CI)**

| Variable | OR (adjusted) | 95% CI (Lower) | 95% CI (Upper) | P-value |
| --- | --- | --- | --- | --- |
| loc_pelvic | 0.067 | 0.006 | 0.757 | 0.029 |
| cyst_yes | 0.023 | 0.001 | 0.370 | 0.008 |
| ratio | 0.001 | 0.000 | 0.273 | 0.017 |
| age | 0.960 | 0.909 | 1.015 | 0.151 |
| male | 0.463 | 0.062 | 3.455 | 0.453 |
| LD | 1.375 | 0.993 | 1.904 | 0.055 |

Abbreviations: CI, confidence interval; OR, odds ratio; LD, long diameter. Values are reported to three decimal places; P<0.001 is reported as '<0.001'. Estimates are from the reduced multivariable logistic regression model after LASSO selection, adjusted for age, sex, and LD. Bootstrap resampling (B=2000) was used for internal validation.

**eTable 3 (extended). Adjusted odds ratios with scaled effect for SD/LD**

| Variable | OR (per +1) | 95% CI (per +1) | OR (per +0.1) | Shrunk OR (per +1) | Shrunk OR (per +0.1) |
| --- | --- | --- | --- | --- | --- |
| SD/LD ratio | 0.001 | 0.000–0.273 | 0.482 (upper CI≈0.878) | 0.040 | 0.724 |

Note: OR(Δ)=exp(β×Δ). The lower CI for the “per +0.1” effect cannot be stably derived because the reported lower CI for the “per +1” effect is rounded to 0.000; the upper CI is approximated as (0.273)^0.1 ≈ 0.878.

**eTable 4. Precision–Recall metrics for the multivariable model and the simplified score**

Note: AP = area under the precision–recall curve (Average Precision). 95% CI via nonparametric bootstrap (n=500). Positive class = 1.

| Model | AP | 95% CI (lower) | 95% CI (upper) |
| --- | --- | --- | --- |
| prob_model_shrunk | 0.937 | 0.836 | 0.991 |
| prob_score_oof | 0.915 | 0.819 | 0.973 |

**eTable 5A. Calibration metrics for the simplified score (5-fold OOF)**

| Metric | Value |
| --- | --- |
| Calibration intercept (OOF) | -0.003 |
| Calibration slope (OOF) | 0.483 |
| Brier score (OOF) | 0.100 |

Note: Event = GN. OOF calibration estimated by logistic regression of outcome on logit(predicted probability).

**eTable 5B. Calibration table (equal-frequency bins, 7 bins; target=deciles)**

| Bin (decile) | n | Predicted prob (mean) | Predicted prob (median) | Observed proportion / Observed positives |
| --- | --- | --- | --- | --- |
| 1 | 8 | 0.001 | 0.001 | 0.000 / 0 |
| 2 | 15 | 0.003 | 0.003 | 0.000 / 0 |
| 3 | 16 | 0.027 | 0.027 | 0.000 / 0 |
| 4 | 15 | 0.207 | 0.207 | 0.400 / 6 |
| 5 | 9 | 0.708 | 0.708 | 0.889 / 8 |
| 6 | 4 | 0.957 | 0.957 | 1.000 / 4 |
| 7 | 7 | 0.995 | 0.995 | 1.000 / 7 |

Note: Event = GN. Equal-frequency bins; due to sample size, some deciles were merged (7 bins in total).

**eTable 6. Confusion matrices at task-oriented thresholds (R1/S1) — Event = GN (for clinical interpretation, exploratory)**

| Model / Strategy | Threshold | TP | FP | TN | FN | Sensitivity | Specificity | PPV | NPV | Total |
| --- | --- | --- | --- | --- | --- | --- | --- | --- | --- | --- |
| Multivariable (shrunk) – S1 (Sp≥0.50) | 0.426 | 23 | 3 | 46 | 2 | 0.920 | 0.939 | 0.885 | 0.958 | 74 |
| Multivariable (shrunk) – R1 (Se≥0.95) | 0.149 | 24 | 8 | 41 | 1 | 0.960 | 0.837 | 0.750 | 0.976 | 74 |
| Simplified score (OOF) – S1 (Sp≥0.50) | 0.594 | 19 | 4 | 45 | 6 | 0.760 | 0.918 | 0.826 | 0.882 | 74 |
| Simplified score (OOF) – R1 (Se≥0.95) | 0.206 | 25 | 20 | 29 | 0 | 1.000 | 0.592 | 0.556 | 1.000 | 74 |

Note: Probabilities from the shrunk multivariable model and 5-fold out-of-fold (OOF) simplified score. Thresholds: Model R1=0.149, S1=0.426; Score R1=0.206, S1=0.594.

**eTable 7. Comparison of equal-weight vs weighted simplified score AUC**

| Model | AUC |
| --- | --- |
| Equal 1-point score | 0.931 |
| Integer-weighted score | 0.940 |

Abbreviations: AUC, area under the curve. Equal 1-point score assigns the same weight (1 point) to each selected ultrasound feature; Integer-weighted score assigns weights based on the rounded regression coefficients. Performance was assessed by the area under the receiver operating characteristic curve.

**eTable 8. Mapping of simplified score to predicted probability**

| Score | n | Observed rate | Predicted probability |
| --- | --- | --- | --- |
| 1 | 9 | 0.000 | 0.003 |
| 2 | 20 | 0.000 | 0.027 |
| 3 | 22 | 0.273 | 0.207 |
| 4 | 10 | 0.600 | 0.708 |
| 5 | 4 | 1.000 | 0.957 |
| 6 | 9 | 1.000 | 0.995 |

Abbreviations: n, number of patients. Observed rate is the proportion of GN cases observed at each score level.

**eTable 9. Optimism-correction metrics for the multivariable logistic regression model**

| Phase | AUC | Brier score | Intercept | Slope |
| --- | --- | --- | --- | --- |
| Apparent | 0.967 | 0.068 | 0.000 | 1.000 |
| Optimism (mean) | 0.037 | -0.033 | -0.040 | 0.558 |
| Corrected | 0.930 | 0.101 | 0.040 | 0.442 |

Abbreviations: AUC, area under the curve. Optimism was estimated from 2000 bootstrap resamples; corrected values represent the apparent performance minus the estimated optimism.

**eTable 10. Original and shrunk coefficients of the multivariable logistic regression model**

| Variable | Coef (original) | Coef (shrunk) | OR (original) | OR (shrunk) |
| --- | --- | --- | --- | --- |
| const | 4.218 | 1.638 | 67.883 | 5.147 |
| loc_pelvic | -2.710 | -1.198 | 0.067 | 0.302 |
| cyst_yes | -3.756 | -1.660 | 0.023 | 0.190 |
| ratio | -7.303 | -3.228 | 0.001 | 0.040 |
| age | -0.040 | -0.018 | 0.960 | 0.982 |
| male | -0.769 | -0.340 | 0.463 | 0.712 |
| LD | 0.318 | 0.141 | 1.375 | 1.151 |

Abbreviations: OR, odds ratio; LD, long diameter. Original coefficients are from the full multivariable model after LASSO selection, adjusted for age, sex, and LD. Shrunk coefficients were obtained by multiplying original coefficients by the calibration slope (0.442) from bootstrap internal validation (B=2000).

**eTable 11. All-strategy threshold table for the multivariable model and simplified score (for clinical interpretation, exploratory)**

| Model | Strategy | Threshold | Sensitivity | Specificity | PPV | NPV | Sensitivity 95% CI (low) | Sensitivity 95% CI (high) | Specificity 95% CI (low) | Specificity 95% CI (high) |
| --- | --- | --- | --- | --- | --- | --- | --- | --- | --- | --- |
| Multivariable | S1: Sp ≥ 0.50 | 0.426 | 0.920 | 0.939 | 0.885 | 0.958 | 0.750 | 0.978 | 0.825 | 0.979 |
| Multivariable | S2: Sp ≥ 0.60 | 0.496 | 0.792 | 0.939 | 0.885 | 0.958 | 0.750 | 0.978 | 0.825 | 0.979 |
| Multivariable | R1: Se ≥ 0.95 | 0.149 | 0.960 | 0.837 | 0.750 | 0.976 | 0.805 | 0.999 | 0.710 | 0.915 |
| Multivariable | R2: rule-in (Sp ≥ 0.80) | 0.426 | 0.920 | 0.939 | 0.885 | 0.958 | 0.750 | 0.978 | 0.825 | 0.979 |
| Simple score | S1: Sp ≥ 0.50 | 0.594 | 0.760 | 0.918 | 0.826 | 0.882 | 0.566 | 0.885 | 0.808 | 0.968 |
| Simple score | S2: Sp ≥ 0.60 | 0.711 | 0.680 | 0.951 | 0.875 | 0.841 | 0.533 | 0.817 | 0.933 | 0.971 |
| Simple score | R1: Se ≥ 0.95 | 0.207 | 1.000 | 0.592 | 0.556 | 1.000 | 0.867 | 1.000 | 0.432 | 0.718 |
| Simple score | R2: rule-in (Sp ≥ 0.80) | 0.594 | 0.760 | 0.918 | 0.826 | 0.882 | 0.566 | 0.885 | 0.808 | 0.968 |

Abbreviations: PPV, positive predictive value; NPV, negative predictive value; Se, sensitivity; Sp, specificity; CI, confidence interval. S1 and R1 correspond to task-oriented thresholds for standard diagnosis (Sp ≥ 0.50) and rule-out (Se ≥ 0.95), respectively. S2 and R2 are alternative strategies with stricter criteria (Sp ≥ 0.60 and Sp ≥ 0.80).

**eTable 12. Multivariable model calculator parameters (shrinkage-adjusted version)**

| Variable | Value (example) | Coefficient | Term |
| --- | --- | --- | --- |
| Intercept |  | 1.638384 | 1.638384 |
| loc_pelvic | 0 | -1.197850 | 0 |
| cyst_yes | 0 | -1.660170 | 0 |
| ratio | 0 | -3.227580 | 0 |
| age | 0 | -0.017820 | 0 |
| male | 0 | -0.340060 | 0 |
| LD | 0 | 0.140702 | 0 |
| Linear predictor |  |  | 1.638384 |
| Predicted probability |  |  | 0.837315 |

Abbreviations: LD, long diameter. Coefficients are from the shrinkage-adjusted multivariable logistic regression model (calibration slope = 0.442 from 2000 bootstrap resamples). Term = Value × Coefficient. Linear predictor = sum of terms. Predicted probability = 1 / (1 + exp(-Linear predictor)). Example values are placeholders; replace with actual patient data.

**eTable 13. Multivariable model calculator parameters (original coefficients)**

| Variable | Value (example) | Coefficient | Term |
| --- | --- | --- | --- |
| Intercept |  | 4.217791 | 4.217791 |
| loc_pelvic | 0 | -2.710330 | 0 |
| cyst_yes | 0 | -3.756400 | 0 |
| ratio | 0 | -7.302910 | 0 |
| age | 0 | -0.040320 | 0 |
| male | 0 | -0.769430 | 0 |
| LD | 0 | 0.318360 | 0 |
| Linear predictor |  |  | 4.217791 |
| Predicted probability |  |  | 0.985483 |

Abbreviations: LD, long diameter. Coefficients are from the original multivariable logistic regression model after LASSO selection, adjusted for age, sex, and LD. Term = Value × Coefficient. Linear predictor = sum of terms. Predicted probability = 1 / (1 + exp(-Linear predictor)). Example values are placeholders; replace with actual patient data.
