# Supplementary figures and images for: Preoperative differentiation of retroperitoneal ganglioneuroma and schwannoma using an ultrasonography-based multivariable model and simplified score: development and single-center internal validation
Source: Front Surg. 2025 Nov 20;12:1685442. doi: 10.3389/fsurg.2025.1685442 (PMC12675405; doi:10.3389/fsurg.2025.1685442)

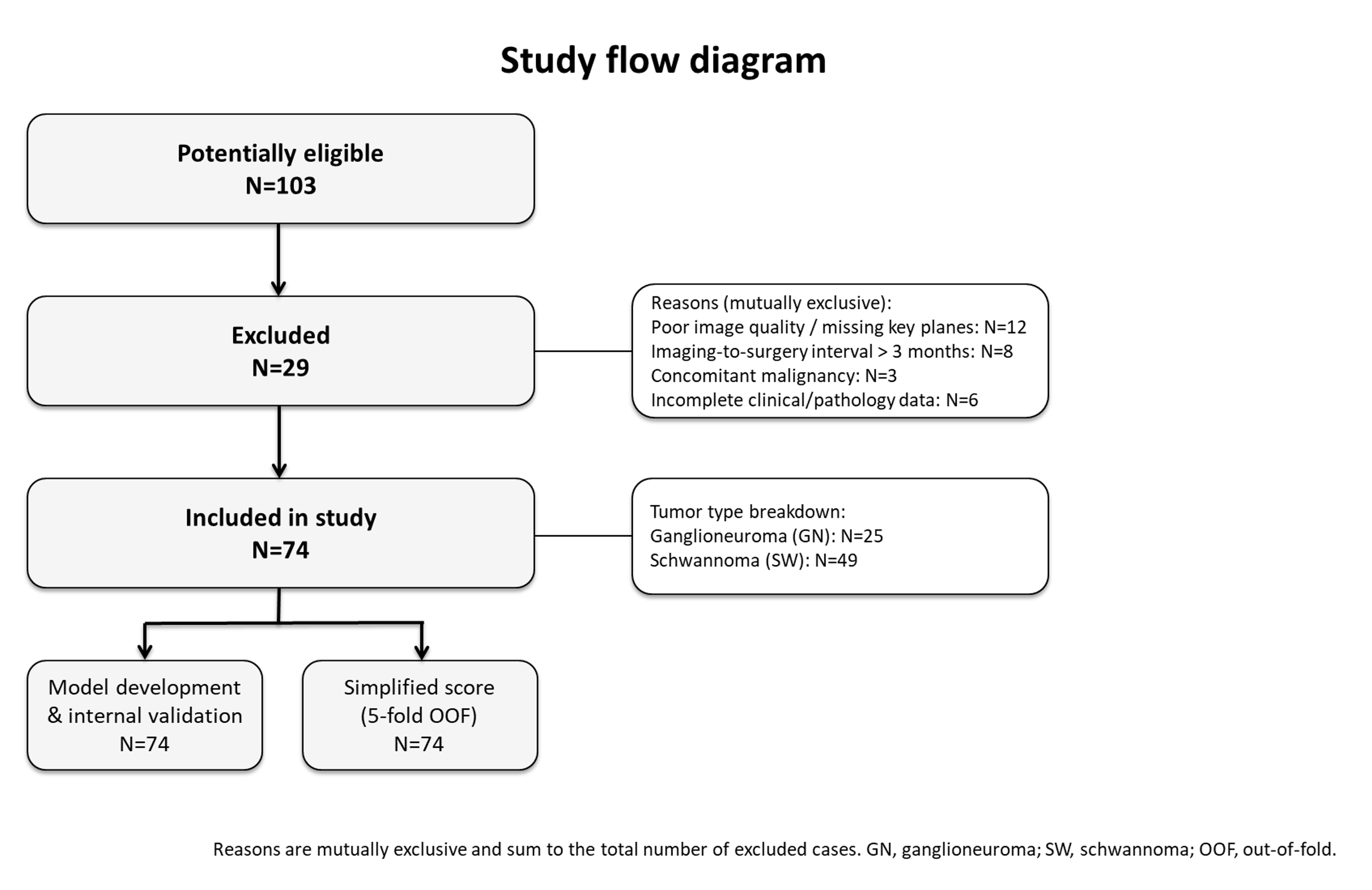

Supplement: Supplementary file 1 [file Supplementaryfile1.zip › eFigure 2.tif]
